# Optimized BCMA/CS1 bispecific TRuC-T cells secreting IL-7 and CCL21 robustly control multiple myeloma

**DOI:** 10.3389/fimmu.2024.1502936

**Published:** 2024-12-24

**Authors:** Min Li, Rong Zheng, Zairu Liu, Peiyuan Zhang, Tingwei Zhu, Xueyi Xin, Hongli Zhao, Wenyi Chen, Binjiao Zheng, Ai Zhao, Jimin Gao

**Affiliations:** ^1^ Key Laboratory of Laboratory Medicine, Ministry of Education, School of Laboratory Medicine and Life Science, Wenzhou Medical University, Wenzhou, China; ^2^ Yicheng County People’s Hospital, Linfen, Shanxi, China; ^3^ Ningbo Hangzhou Bay Hospital, Ningbo, Zhejiang, China; ^4^ Taizhou Hospital of Zhejiang Province Affiliated to Wenzhou Medical University, Taizhou, Zhejiang, China; ^5^ Taizhou Central Hospital (Taizhou University Hospital), Taizhou, Zhejiang, China; ^6^ Affiliated Hangzhou First People’s Hospital, Westlake University School of Medicine, Hangzhou, Zhejiang, China; ^7^ Zhejiang Qixin Biotech, Wenzhou, Zhejiang, China

**Keywords:** multiple myeloma, T cell receptor fusion construct T cell, B cell maturation antigen, CD2 subset 1, interleukin-7, C-C motif chemokine ligand 21

## Abstract

**Introduction:**

Challenges remain in reducing antigen escape and tumor recurrence while CAR-T cell therapy has substantially improved outcomes in the treatment of multiple myeloma. T cell receptor fusion construct (TRuC)-T cells, which utilize intact T cell receptor (TCR)-CD3 complex to eliminate tumor cells in a non-major histocompatibility complex (MHC)-restricted manner, represent a promising strategy. Moreover, interleukin-7 (IL-7) is known to enhance the proliferation and survival of T cells. C-C motif chemokine ligand 21 (CCL21) is a ligand for chemokine C-C motif receptor 7 (CCR7) and exhibits strong chemotaxis against naïve T cells and antigen-presenting cells such as dendritic cells.

**Methods:**

The bispecific TRuC-T cells simultaneously targeting B cell maturation antigen (BCMA) and CD2 subset 1 (CS1) were constructed by pairing two of five subunits (i.e., TCRαC, TCRβC, CD3γ, CD3δ, and CD3ϵ) in the TCR/CD3 complex and were named C-AC-B-3E, C-BC-B-3E, C-3G-B-3E, C-3D-B-3E, C-3E-B-3E, B-3E-C-3E, B-3G-C-3E, and B-3D-C-3E. Additionally, the BCMA/CS1 bispecific TRuC-T cells secreting IL-7 and CCL21, named BC-7×21 TRuC-T cells, were generated. All of the bispecific TRuC-T cells were characterized and tested *in vitro* and *in vivo*.

**Results:**

Following the optimization of various pairs of two subunits of TCR/CD3 complex, B-3G-C-3E TRuC-T cells, characterized by incorporating CD3γ and CD3ε, exhibited the strongest myeloma-specific cytotoxicity. Furthermore, the bispecific BC-7×21 TRuC-T cells had stronger proliferation, chemotaxis, and cytotoxicity *in vitro*. Accordingly, the bispecific BC-7×21 TRuC-T cells showed better persistence in vivo so as to effectively suppress tumor growth in the NCG mouse xenograft model of MM.1S multiple myeloma.

**Discussion:**

This study demonstrated that BC-7×21 TRuC-T cells, engineered through the optimization of the two subunits of TCR/CD3 complex and a co-expression cytokine strategy, may offer a novel and effective therapy for relapsed/refractory multiple myeloma.

## Introduction

Multiple myeloma is a plasma cell malignancy caused by the abnormal proliferation of clonal plasma cells in the bone marrow to produce monoclonal immunoglobulins and/or light chains, resulting in end-organ damage (1). Monoclonal antibodies, proteasome inhibitors, and immunomodulators provide new ideas for the treatment of multiple myeloma. However, multiple myeloma is prone to recurrence and remains predominantly incurable, especially for high-risk patients who do not benefit from these therapies (2, 3).

The history of chimeric antigen receptor-T (CAR-T) cell therapy dates back three decades, and the field has rapidly evolved from the first-generation to the fifth-generation of CARs (4–7). Over these years, CAR-T cell therapy has been proven to be highly effective in treating hematological malignancies (8–10). Two US Food and Drug Administration (FDA)approved CAR-T cell products targeting B cell maturation antigen (BCMA) for the treatment of relapsed/refractory multiple myeloma, Idecabtagene vicleucel (Ide-cel, bb2121, Celgene/BMS) and Ciltacabtagene autoleucel (Ciltacel, JNJ-68284528/LCAR-B38M, Janssen), have provided a new strategy to some extent. However, CAR-T cell immunotherapies for relapsed/refractory multiple myeloma are mainly of the second-generation, which are prone to lead to cytokine release syndrome (CRS) and neurotoxicity after treatment (11, 12).

Unlike CAR-T cells, T cell receptor (TCR)-T cells target solid tumors primarily by recognizing intracellular tumor antigens presented by major histocompatibility complex (MHC) and engaging in CD3 signaling mechanisms (13). The TCR/CD3 complex comprises a peptide-MHC ligand-binding domain, consisting of a TCRα and a TCRβ chains, along with a CD3 signaling domain. The CD3 signaling domain mainly includes dimers of CD3ϵ and CD3γ, dimers of CD3ϵ and CD3δ, as well as CD3ζ homodimers (14, 15). Original CAR structures utilize only the intracellular signaling domain of the CD3ζ chain, which is isolated from other five subunits of the TCR/CD3 complex.

T cell receptor fusion constructs (TRuCs) contain antibody-based binding domains fused to the TCR/CD3 complex subunit and become a functional component of the TCR/CD3 complex, enabling efficient reprogramming of the intact TCR/CD3 complex to recognize tumor cell surface antigens. TRuC-T cells kill tumor cells as effectively as CAR-T cells, but with lower cytokine release (16, 17).

BCMA, a member of the tumor necrosis factor receptor superfamily 17 (TNFRSF17), is preferentially expressed on plasma cells but not on CD34^+^ hematopoietic stem cells, making it a promising therapeutic target (18). However, the heterogeneity of BCMA expression on multiple myeloma cells allows anti-BCMA CAR-T cells to preferentially target multiple myeloma cells with high BCMA expression, while to retain multiple myeloma cells with low or no BCMA expression for clonal growth (19–21). Multiple myeloma cells typically lose BCMA when the disease recurs after anti-BCMA CAR-T cell infusion. This indicates that the CAR-T cells have selected BCMA-negative multiple myeloma cell clones (22–25). Therefore, dual-antigen targeting CAR-T therapies have been evaluated in multiple early-stage clinical trials to improve response rates and prevent relapse. A variety of strategies can be employed to target multiple antigens with CAR-T therapy, including co-administration of various CAR-T products, the use of bicistronic or tandem CARs, or co-transduction with different CAR constructs (26).

CD2 subset 1 (CS1), also known as signaling lymphocyte activation molecule family member 7 (SLAMF7), is a glycosylated cell surface protein that belongs to the signaling lymphocyte activation molecule (SLAM) family. CS1 plays an important role in the adhesion of myeloma cells to bone marrow stromal cells (27).

To address these challenges, we are committed to developing new therapies for relapsed/refractory multiple myeloma that exhibit both greater resistance to antigen escape and long-term anti-tumor effects. In this work, we combined five subunits of TCRαC, TCRβC, CD3γ, CD3δ, and CD3ϵ in the TCR/CD3 complex to construct BCMA/CS1 bispecific TRuC-T cells, named C-AC-B-3E, C-BC-B-3E, C-3G-B-3E, C-3D-B-3E, C-3E-B-3E, B-3E-C-3E, B-3G-C-3E, and B-3D-C-3E. Interleukin-7 (IL-7) is known to enhance the proliferation and survival of T cells (28–30). C-C Motif Chemokine Ligand 21 (CCL21) is a ligand for chemokine C-C motif receptor 7 (CCR7) and exhibits strong chemotaxis against naïve T cells and antigen-presenting cells (APCs) such as dendritic cells (DCs) (31, 32). Additionally, we prepared BCMA/CS1 bispecific TRuC-T cells secreting IL-7 and CCL21, named BC-7×21 TRuC-T cells. We found that the C-3G-B-3E, C-3D-B-3E, B-3G-C-3E, and B-3D-C-3E bispecific TRuC-T cells prepared by the combination of either CD3γ-CD3ϵ or CD3δ-CD3ϵ had high expression levels of TRuCs on T cells that could efficiently kill multiple myeloma cells. In the NCG mouse xenograft model, B-3G-C-3E TRuC-T cells exhibited stronger anti-tumor effects than B-3D-C-3E TRuC-T cells. Furthermore, BC-7×21 TRuC-T cells were substantially more effective than B-3G-C-3E TRuC-T cells in improving the growth of TRuC-T cells *in vitro*, leading to a persistence of TRuC-T cells in tumor-bearing mice. Thus, the novel platform may provide a promising strategy for the treatment of multiple myeloma.

## Materials and methods

### Cell lines maintenance

U266, MM.1S, IM9, Raji, and K562 cells were conserved by our laboratory. HEK-293T cells were purchased from the Chinese Academy of Sciences (Shanghai, China). Raji and K562 cells were transduced to stably express human BCMA or CS1 with lentivirus (designated as BCMA-Raji, CS1-K562). HEK-293T cells were cultivated in DMEM medium (Sigma-Aldrich, St. Louis, MO, USA) supplemented with 10% FBS (PAN Biotech, Adenbach, Germany). U266, MM.1S, IM9, Raji, and K562 cells were cultivated in RPMI 1640 (Sigma-Aldrich) supplemented with 10% FBS.

### Plasmid construction and TRuC-T cells generation

The BCMA or CS1 TRuCs consist of an anti-human BCMA single chain variable fragment (scFv) or an anti-human CS1 scFv tandem with the human TCRαC, TCRβC, CD3γ, CD3δ, or CD3ϵ domains via a linker (G4S)4. Anti-BCMA-CD3E TRuC was combined with CS1-TCRαC, CS1-TCRβC, CS1-CD3G, CS1-CD3D, or CS1-CD3E using a 2A self-cleaving peptide to construct BCMA/CS1 bispecific TRuCs lentiviral vectors: C-AC-B-3E, C-BC-B-3E, C-3G-B-3E, C-3D-B-3E, and C-3E-B-3E. B-3G-C-3E TRuC was conjugated with IL-7 and CCL21 by 2A peptide to form BC-7×21 TRuC. BCMA or CS1 scFv combined into a CAR with fusion to CD8a hinge and transmembrane region and the intracellular signaling domains of human 4-1BB and CD3ζ motif in tandem. Then all TRuCs and CARs were cloned into the pLenti-CMV-Puro vector to obtain the recombinant plasmid.

HEK-293T cells were transfected with TRuC-expressing recombinant plasmid together with the lentiviral packaging plasmid pLP1, pLP2, and pMD2G by using polyethyleneimine (Polysciences, Inc. Warrington, PA, USA). Lentiviral supernatant was collected and lentiviral particles were concentrated 400-fold by horizontal centrifugation. Peripheral blood mononuclear cells (PBMCs) were isolated from whole blood of healthy donors by Ficoll density gradient centrifugation. T cells were enriched with anti-human CD3/CD28 beads (Invitrogen, Carlsbad, CA, USA) and stimulated for 24 h in the KBM581 serum-free medium (Corning, NY, USA) supplemented with IL-2 (20 IU/ml, Peprotech, Rocky Hill, NJ, USA). Activated T cells were infected with lentivirus at multiplicity of infection (MOI)=40.

### Flow cytometry

Human PE-BCMA and FITC-BCMA recombinant proteins (ACRO Biosystems, Newark, USA) were used to detect the expression of anti-BCMA scFvs. Human CS1-Biotinylated recombinant protein (ACRO Biosystems) was used to detect the expression of anti-CS1 scFvs and then followed by streptavidin with APC fluorescein. Anti-human CCR7 antibody (PE), anti-human CD45RO antibody (FITC), anti-human CD45RA antibody (APC), anti-human CD8α antibody (PE-Cy7), and anti-human CD4 antibody (APC-Cy7) were used to detect TRuC-T cell subtypes. Anti-human PD1 antibody (APC), anti-human LAG3 antibody (PE), and anti-human TIM3 antibody (APC) were used to detect TRuC-T cell exhaustion. Anti-human CD69 antibody (PE-Cy7) and anti-human CD25 antibody (APC) were used to detect TRuC-T cell activation and tonic signaling. Anti-human CD3 antibody (PE-Cy7) was used to detect human T cells in NCG mice. All antibodies were purchased from BioLegend (San Diego, CA, USA). Data were analyzed with FlowJo 10 (FlowJo, USA).

### Western blot

After TRuC-T cell lysis, protein samples were separated in 10% SDS-PAGE and transferred onto PVDF membrane (Bio-Rad, Hercules, CA, USA). Membranes were blocked in 5% skimmed milk powder solution for 1 h at room temperature, followed by overnight incubation at 4°C with rabbit anti-human CD3γ, CD3δ, CD3ϵ, or CD3ζ antibodies (Abcam, Cambridge, UK), Washed with TBS-Tween 20, incubated with HRP-conjugated goat anti-rabbit IgG (H&L) antibody (Beyotime, Shanghai, China) for 2 h at room temperature. Chemiluminescence solution (BioVision, San Francisco, CA, USA) was added on membrane followed by image scan using imaging lab ™ software (Bio-Rad).

### Enzyme-linked immunosorbent assay

Cell culture supernatant of BC-7×21 TRuC-T cells was collected on day 7 to detect the IL-7 and CCL21 by ELISA kit (MULTISCIENCES, Hangzhou, ZJ, China). Mock-T and TRuC-T cells were co-incubated with U266 cells at an effector-to-target (E: T) ratio of 1:1 for 24 h and supernatants were collected. ELISA kits were used to detect IL-2 and IFN-γ (MULTISCIENCES).

### Proliferation analysis and apoptosis assay

BC-7×21 TRuC-T cells were labeled with CellTrace™ carboxyfluorescein diacetate succinimidyl ester (CFSE, Invitrogen) and the mean fluorescence intensity (MFI) was detected by flow cytometry. Annexin V/7AAD apoptosis detection kit (BioLegend) was used to determine the survival of TRuC-T cells.

### Cell migration assay

T cells labeled with CellTrace™ CFSE were added to the upper chamber of a 5-μm pore size polycarbonate filter transwell (Corning). BC-7×21 TRuC-T cell culture supernatant was collected and placed in the lower chambers. After 4 h incubation, CFSE-labeled T cells migrating into the lower chamber were observed with a fluorescence microscope and taken pictures randomly. The CFSE-labeled T cells migrating from the upper chamber to the lower chamber were counted.

### *In vitro* cytotoxicity assay

Luciferase-expressing tumor cells were plated in triplicates in a 96-well plate with 10000 cells per well and T cells were added at the desired E: T ratios. Tumor cells added with double distilled water served as a positive control (Kmax), and tumor cells added with complete medium served as a negative control (Kmin). After 8 h culture, 0.5 mM D-luciferin (Sigma-Aldrich) was added to each well, and the fluorescence intensity was measured by luminometric measurement on a microplate reader after 10 min. The percentage of tumor lysis was calculated as following formula: lysis (%) = (Kmin – K)/(Kmin – Kmax) × 100%.

### Animal experiments

All animal studies were approved by the Laboratory Animal Ethics Committee of Wenzhou Medical University. All NCG mice were housed under specific pathogen-free conditions at the Wenzhou Medical University Experimental Animal Center (Wenzhou, ZJ, China).

6-to-8-week-old female NCG (NOD-Prkdcem26Cd52IL-2rgem26Cd22/Nju) mice were purchased from Gem Pharmatech Co. Ltd (Nanjing, JS, China). On day 0, 1.0×10^6^ MM.1S-Luc cells were injected into the tail veins of NCG mice, and the mice were randomly divided into three groups (*N* = 3 mice per group). TRuC-T cells were injected intravenously (i.v.) on day 6.

In the rechallenged model, 6-to-8-week-old female NCG mice were randomly divided into five groups (*N* = 6 mice per group). When multiple myeloma was controlled, NCG mice were injected i.v. with 1.0×10^6^ MM.1S-Luc cells. Treatment with Mock-T cells served as a negative control. Tumor progression was monitored by bioluminescence imaging using an IVIS imaging system (PerkinElmer, Shanghai, China), and the intensity of MM.1S-Luc cells signal was measured as total photon/second/cm^2^/steradian(p/sec/cm^2^/sr). To assess histopathological changes, tissues were fixed with 4% paraformaldehyde and embedded in paraffin. The tissues were sliced into 4-μm thick sections and then stained with hematoxylin/eosin (H&E) for visualization of tissue structure.

### Statistical analysis

All data were analyzed using GraphPad Prism 9.0 software (La Jolla, CA, USA). Statistical analysis was performed using unpaired two-tailed Student’s t-test or ANOVA and multiple comparisons were made using Bonferroni’s correction. All experiments were repeated at least three times. *P*-values < 0.05 were considered statistically significant and indicated as follows: ns, no significant difference, *P < 0.05, **P < 0.01, ***P < 0.001, ****P < 0.0001.

## Results

### Preparation of BCMA/CS1 bispecific TRuC-T cells

**Figure 1:**
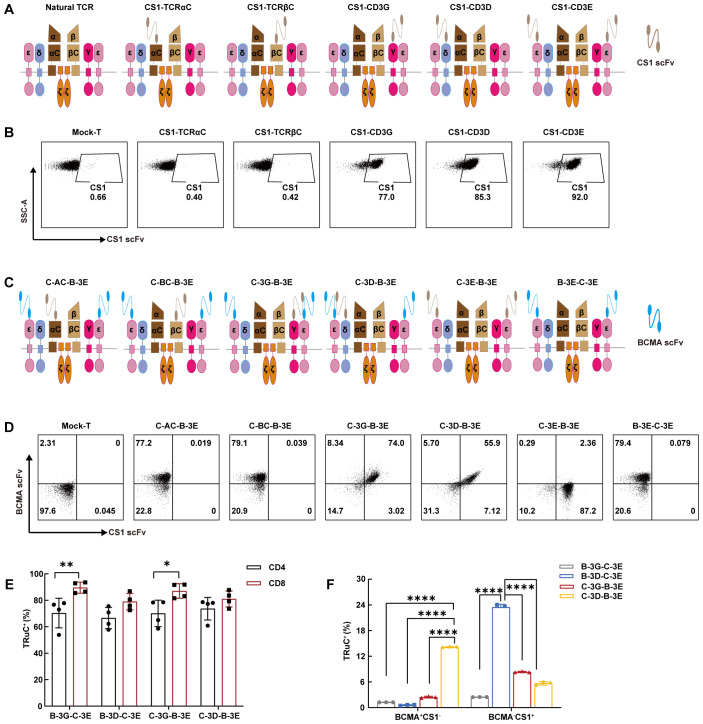
Bispecific TRuC-T cells linked to CD3δ and CD3ϵ subunits exhibited unstable integration efficiency. **(A)** Schematic illustration of CS1 single-target TRuCs and natural αβTCR. **(B)** Flow cytometry was used to detect surface expression level of CS1 single-target TRuCs with human CS1-Biotinylated recombinant proteins. **(C)** Schematic illustration of BCMA/CS1 bispecific TRuCs. **(D)** The surface expression level of BCMA/CS1 bispecific TRuCs on T cells. **(E)** The expression level of four bispecific TRuCs in CD4^+^ T subsets and CD8^+^ T subsets. Statistical analysis diagram was illustrated (*N* = 4). **(F)** Flow cytometry was used to detect the expression of bispecific TRuCs on T cells cultured *in vitro* for 16 days (*N* = 3). Statistical analysis values are shown as mean values ± SD. The data shown are representative of results from at least three independent experiments performed with cells from at least three different healthy donors. *P*-values in **(E)** were calculated by unpaired two-tailed Student’s t-test. *P*-values in **(F)** were calculated by one-way ANOVA. Multiple comparisons were made using Bonferroni’s correction. ANOVA, analysis of variance; *P < 0.05, **P < 0.01, ****P < 0.0001.

We fused scFv targeting human CS1 with human TCRαC, TCRβC, CD3γ, CD3δ, or CD3ϵ into CS1 single-target TRuCs via linker (G4S)4 and designated them as CS1-TCRαC, CS1-TCRβC, CS1-CD3G, CS1-CD3D and CS1-CD3E, respectively (**Figure 1A**; **Supplementary Figure 1A**). Then peripheral blood T cells from healthy donors were infected with the indicated lentiviruses. Low-level expression of CS1 single-target TRuCs on T cells was detected in CS1-TCRαC and CS1-TCRβC TRuC-T cells, while CS1-CD3G, CS1-CD3D, and CS1-CD3E TRuC-T cells had high-level expression of CS1 single-target TRuCs (**Figure 1B**). The MFI of CS1 single-target TRuCs in CS1-CD3E TRuC-T cells was the highest (**Supplementary Figure 1B**). These findings were consistent with the observations from TRuCs targeting human BCMA (**Supplementary Figures 1C, D**). The 2A self-cleaving peptide was used to create various BCMA/CS1 bispecific TRuC constructs by pairing BCMA-CD3E TRuC with different CS1 components, such as CS1-TCRαC, CS1-TCRβC, CS1-CD3G, CS1-CD3D, or CS1-CD3E (**Figure 1C**; **Supplementary Figure 1E**). Then BCMA/CS1 bispecific TRuCs expression level on the surface of T cells was detected by flow cytometry (**Figure 1D**). BCMA scFvs were highly expressed on C-AC-B-3E and C-BC-B-3E TRuC-T cells, whereas CS1 scFvs were low or absent. Both BCMA scFvs and CS1 scFvs could efficiently integrate into the TCR/CD3 complex of C-3G-B-3E and C-3D-B-3E TRuC-T cells. CS1 scFvs exhibited a little expression on C-3E-B-3E TRuC-T cells, whereas BCMA scFvs were hardly expressed. Similarly, B-3E-C-3E TRuC-T cells had only BCMA scFvs expression. The results suggested that only the CD3ϵ-TRuC at the upstream of the expression vector could integrate into the TCR/CD3 complex so as to be expressed on the surface of T cells. Two combinations of CD3γ-CD3ϵ and CD3δ-CD3ϵ were assessed in subsequent experiments to discern the distinctions among four bispecific BCMA/CS1 TRuC-T cell constructs: C-3G-B-3E, C-3D-B-3E, B-3G-C-3E, and B-3D-C-3E. Flow cytometric analysis revealed a lower expression efficiency of the above four TRuC constructs in CD4^+^ T subsets than that in CD8^+^ T subsets, especially for B-3G-C-3E and C-3G-B-3E TRuC-T cells (**Figure 1E**). Western-Blot showed 55 kDa recombinant protein molecules with anti-BCMA CAR-T (33) served as a negative control (**Supplementary Figure 1F**). The B-3D-C-3E and C-3D-B-3E TRuCs showed instability, as indicated with a higher frequency of single-positive cells after 16 days of *in vitro* culture. (**Figure 1F**).

### Characteristics of BCMA/CS1 bispecific TRuC-T cells *in vitro*

**Figure 2:**
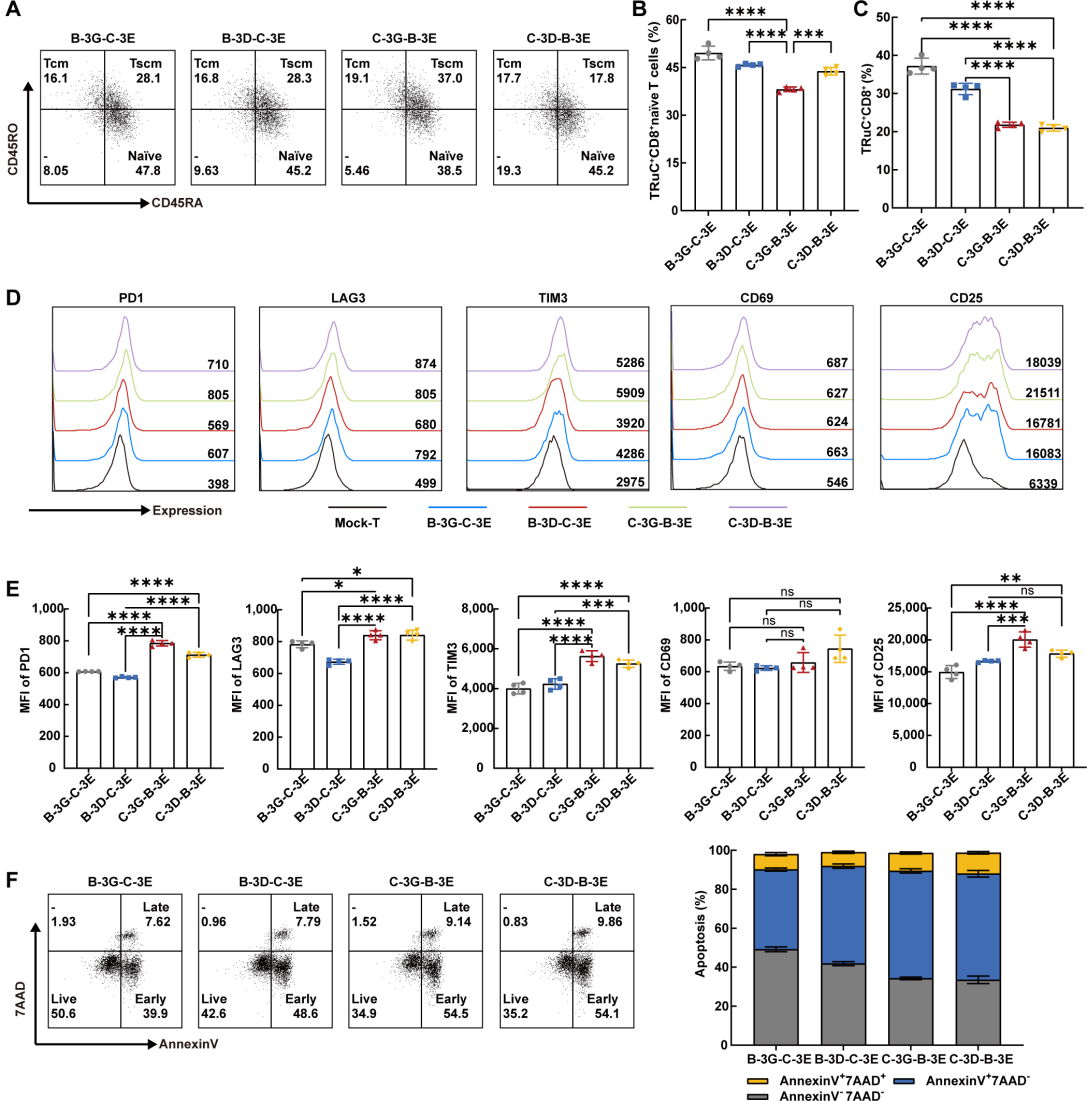
Bispecific TRuC-T cells showed phenotypic heterogeneity *in vitro*. **(A)** The expression of CD45RO and CD45RA in CD8^+^CCR7^+^ T cells were measured by flow cytometry. Representative flow cytometric result was illustrated. Naïve T cells (CCR7^+^CD45RA^+^CD45RO^-^, naïve), central memory T cells (CCR7^+^CD45RA^-^CD45RO^+^, Tcm), stem cell memory T cells (CCR7^+^CD45RA^+^CD45RO^+^, Tscm). **(B)** The proportion of CD8^+^naive T subsets in four bispecific TRuC-T cells (*N* = 4). **(C)** Ratios of CD8^+^T phenotype in four bispecific TRuC-T cells (*N* = 4). **(D, E)** The expression level of PD1, LAG3, TIM3, CD69 and CD25 on Mock-T and four bispecific TRuC-T cells. Representative pictures **(D)** and statistical analysis diagram **(E)** were illustrated (*N* = 4). **(F)** Four bispecific TRuC-T cells were stained with Annexin V/7AAD and the apoptosis was detected by flow cytometry. Representative pictures (left panel) and statistical analysis diagram (right panel) were shown. Data are presented as mean values ± SD. The data shown are representative of results from at least three independent experiments performed with cells from at least three different healthy donors. *P*-values in **(B, C, E)** were calculated by one-way ANOVA. Multiple comparisons were made using Bonferroni’s correction. ns, no significant difference, *P < 0.05, **P < 0.01, ***P < 0.001, ****P < 0.0001.

It has shown that effector cells derived from naïve CD8^+^ T cells exhibit greater cytotoxic capabilities than those from central memory T cells (34). We examined the CD4/CD8 ratio and cell subtype of four BCMA/CS1 bispecific TRuC-T cells. Notably, flow cytometric results showed that the CD8^+^ naïve T subset proportion in C-3G-B-3E TRuC-T cell was the lowest (**Figures 2A, B**). C-3G-B-3E and C-3D-B-3E TRuC-T cells had fewer CD8^+^T cells than those of B-3G-C-3E and B-3D-C-3E TRuC-T cells (**Figure 2C**). In addition to multiple myeloma cells, CS1 is expressed at low levels on other hematopoietic cells such as CD8^+^ T cells (35, 36).The non-specificity of the antigen may result in a poorer response to treatment of myeloma cells. Furthermore, the proportion of CD8^+^ T cells in anti-CS1 CAR-T cells was lower than that of CS1-CD3G, CS1-CD3D, and CS1-CD3E TRuC-T cells, indicating lower fratricide propensity by CS1 TRuC-T cells than anti-CS1 CAR-T cells (**Supplementary Figure 2A**).

The exhaustion and survival of CAR-T cells are essential for the persistence of CAR-T therapy *in vivo (* 37). The exhausted T cell phenotype is typically characterized by increased expression of several inhibitory receptors, such as programmed cell death protein 1 (PD1), lymphocyte activation gene 3 (LAG3), and T-cell immunoglobulin 3 (TIM3) (38). Utilizing flow cytometry, we assessed the levels of PD1, LAG3, and TIM3, alongside Annexin V/7AAD to ascertain immune checkpoint expression and apoptosis of four BCMA/CS1 bispecific TRuC-T cells, respectively. The results depicted that the immune checkpoints expression level of B-3G-C-3E and B-3D-C-3E TRuC-T cells were lower than that of C-3G-B-3E and C-3D-B-3E TRuC-T cells (**Figures 2D, E**). The C-3D-B-3E TRuC-T cells had a significantly reduced survival rate compared with B-3G-C-3E and B-3D-C-3E TRuC-T cells (**Figure 2F**). Considering that tonic signaling from CAR, the spontaneous CAR activation in the absence of tumor antigen stimulation, plays a crucial role in controlling CAR-T efficacy (39), we measured activation markers CD69 and CD25 and found that the MFI of CD25 was significantly higher in the C-3G-B-3E TRuC-T cells than in the B-3G-C-3E and B-3D-C-3E TRuC-T cells (**Figures 2D, E**).

### BCMA/CS1 bispecific TRuC-T cells showed effective cytotoxicity *in vitro* and *in vivo*

**Figure 3:**
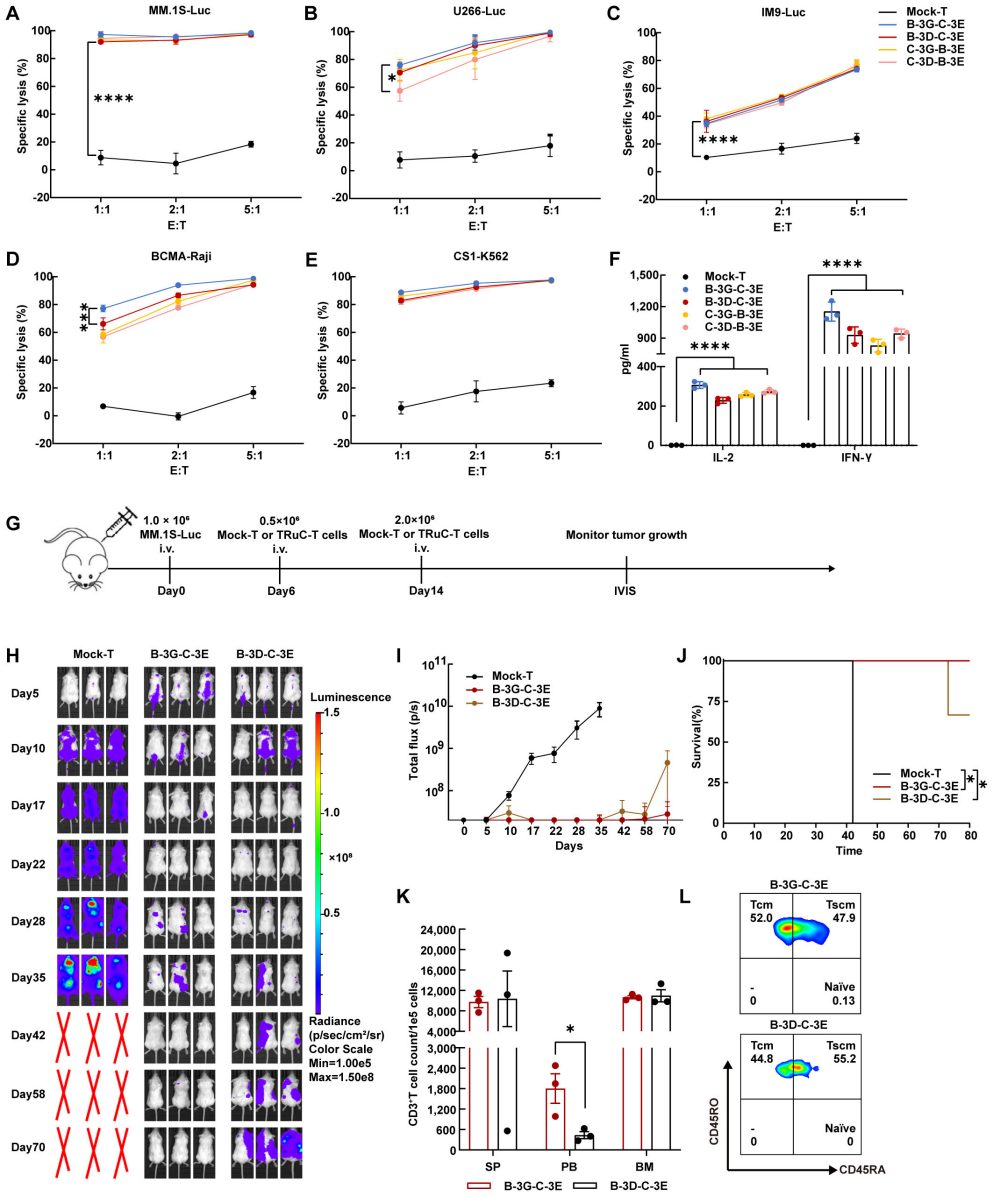
BCMA/CS1 bispecific TRuC-T cells linked to CD3γ and CD3ϵ subunits owned stronger killing capacity against multiple myeloma *in vivo*. **(A–E)**. Cell-lysis activity of BCMA/CS1 bispecific TRuC-T cells against MM.1S, U266, IM9, BCMA-Raji, and CS1-K562 cells at E: T ratios of 1:1, 2:1, and 5:1. The specific lysis (%) was quantified after an 8 h coincubation (*N* = 3). **(F)**. *In vitro* cytokine analysis of supernatants from co-culture of four bispecific TRuC-T cells with U266 cells (E: T=1:1) for 24 h (*N* = 3). **(G)** Treatment scheme for MM.1S-luc tumor-bearing mice, intravenously (i.v.). **(H)** Mice were engrafted with 1.0 × 10^6^ MM.1S-Luc cells and then treated with 0.5 × 10^6^ Mock-T or TRuC-expressing T cells on day 6 and 2.0 × 10^6^ Mock-T or TRuC-expressing T cells on day 14. Tumor progression was monitored by bioluminescence imaging. **(I)** Bioluminescence kinetics in each group of mice (*N* = 3). **(J)** Kaplan-Meier survival curve. Log-rank tests were used (*N* = 3). **(K)** The count of human CD3^+^ T cells in the spleen (SP), peripheral blood (PB) and bone marrow (BM) of mice after the treatment with B-3G-C-3E or B-3D-C-3E bispecific TRuC-T cells (*N* = 3). **(L)** T cell subtypes in the peripheral blood of mice treated with B-3G-C-3E or B-3D-C-3E bispecific TRuC-T cells. Data are presented as mean values ± SD in **(A–F)** and mean values ± SEM in **(I, K)**. *P*-values in **(A–D)** were calculated by two-way ANOVA. One-way ANOVA was used in **(F)** and unpaired two-tailed Student’s t-test was used in **(K)**. Multiple comparisons were made using Bonferroni’s correction. *P < 0.05, ***P < 0.001, ****P < 0.0001.

We established multiple myeloma cell lines expressing luciferase (Luc) and detected their expression of BCMA and CS1 antigen (**Supplementary Figure 2B**). The cytotoxicity of four bispecific TRuC-T cells against several cell lines: MM.1S (BCMA^+^CS1^+^) cells, U266 (BCMA^+^CS1^+^) cells, IM9(BCMA^+^CS1^+^) cells, BCMA-Raji (BCMA^+^CS1^-^) cells, and CS1-K562 (BCMA^-^CS1^+^) cells were assessed (**Figures 3A–E**). To mimic the mixed expression of antigen on multiple myeloma, BCMA-Raji and CS1-K562 cells were mixed at different ratios (**Supplementary Figure 2C**). All four bispecific TRuC-T cells demonstrated significant cytotoxicity which increased with higher E: T ratios. At an E: T ratio of 1:1, B-3G-C-3E TRuC-T cells exhibited better killing ability, particularly against BCMA-Raji and U266 cells. However, no differences in the killing ability were observed among the four bispecific TRuC-T cells with the increase of E: T ratios. After 24 h incubation with U266 cells (E: T=1:1), all four bispecific TRuC-T cells secreted higher levels of IL-2 and IFN-γ than Mock-T cells (**Figure 3F**).

Intravenous injection of 1.0 × 10^6^ MM.1S-Luc cells into NCG mice established a multiple myeloma model by day 5 which was confirmed by bioluminescence imaging. On day 6, NCG mice were randomly grouped and injected with 0.5 × 10^6^ Mock-T, B-3G-C-3E, and B-3D-C-3E bispecific TRuC-T cells. Furthermore, a second injection of 2.0 × 10^6^ corresponding T cells was administered on day 14 (**Figure 3G**). By day 28, mice in the Mock-T group had large tumor burdens, while tumor remission occured in the mice of B-3G-C-3E and B-3D-C-3E bispecific TRuC-T groups. On day 42, multiple myeloma in mice treated with B-3G-C-3E bispecific TRuC-T cells was almost in complete remission, but multiple myeloma recurred in the B-3D-C-3E bispecific TRuC-T group (**Figures 3H, I**). Eventually, one mouse from the B-3D-C-3E TRuC-T group suffered hind limb paralysis and died (**Figure 3J**). There was no significant change of body weight in each group of mice (**Supplementary Figure 2D**). We detected the relapsed tumor cells in the bone marrow of B-3D-C-3E TRuC-T treated mouse and found that the MM.1S-Luc cells retained antigen expression (**Supplementary Figure 2E**). Significantly higher presence frequencies of human CD3^+^ T cells were detected in the peripheral blood of mice treated with B-3G-C-3E TRuC-T cells (**Figure 3K**). Besides, flow cytometric analysis showed that mainly memory T cells persisted in mice after the B-3G-C-3E and B-3D-C-3E bispecific TRuC-T cells attacked the multiple myeloma (**Figure 3L**).

### Autocrine production of IL-7 and CCL21 enhanced the proliferation and chemotaxis of BCMA/CS1 bispecific TRuC-T cells

**Figure 4:**
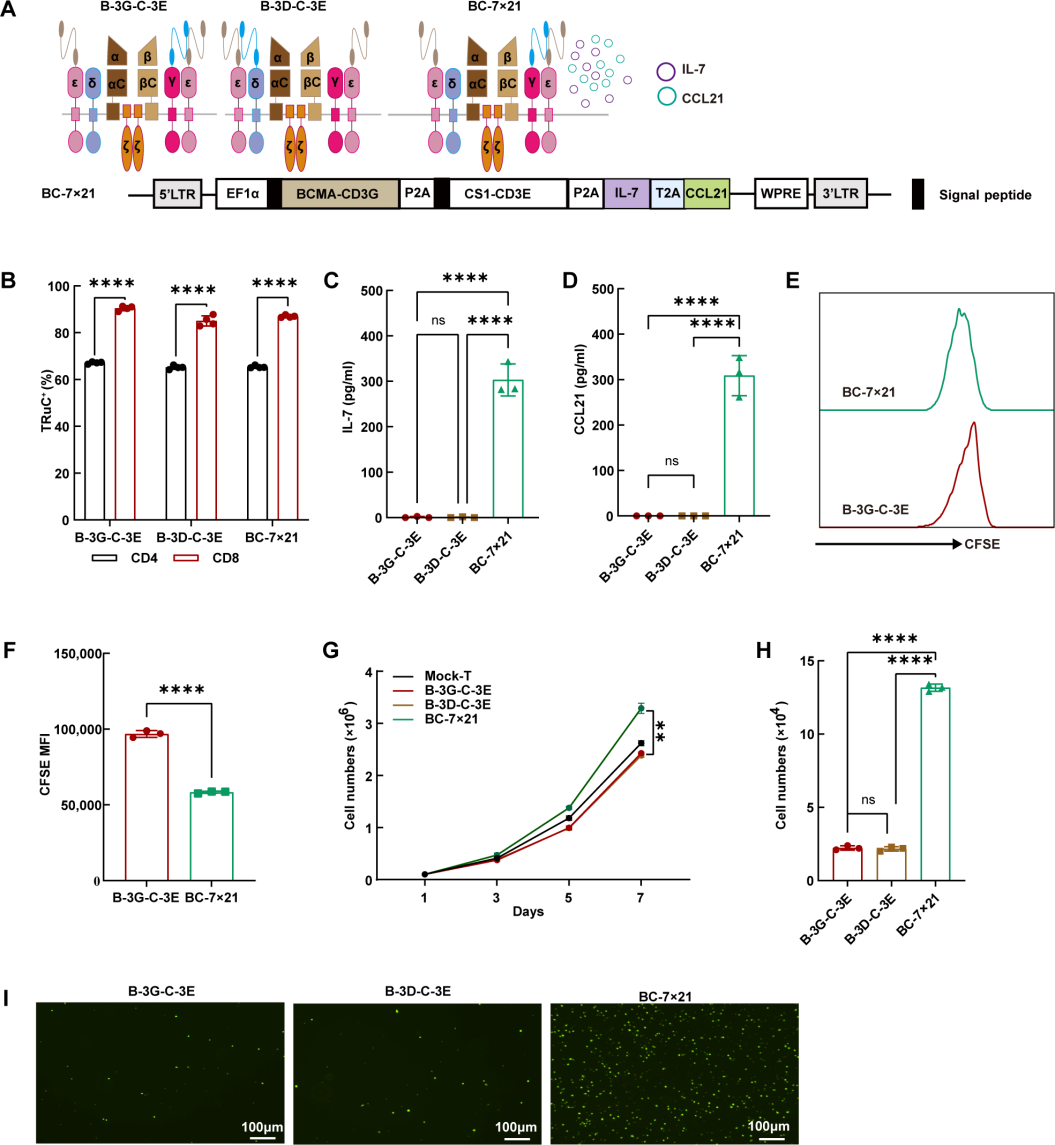
The effective secretion of IL-7 and CCL21 by BC-7×21 TRuC-T cells. **(A)** Schematic illustration (upper panel) and engineered domain architecture (lower panel) of the BC-7×21 TRuC structure. **(B)** The expression of bispecific TRuCs in CD4^+^ T subsets and CD8^+^ T subsets of B-3G-C-3E, B-3D-C-3E and BC-7×21 TRuC-T cells. Statistical analysis diagram was shown (*N* = 4). **(C, D)** The cell culture supernatants of conventional BCMA/CS1 bispecific TRuC-T and BC-7×21 TRuC-T were detected for concentrations of IL-7 **(C)** and CCL21 **(D)** by ELISA (*N* = 3). **(E, F)** B-3G-C-3E and BC-7×21 TRuC-T cells were labeled with CFSE, and the dilution of CFSE was determined by flow cytometry after 5 days (*N* = 3). **(G)** Proliferative capacity of TRuC-T cells was tested by counting (*N* = 4). **(H, I)** Transwell assays were used to verify the chemotactic effect of CCL21 secreted from BC-7×21 TRuC-T cells (*N* = 3). Data are presented as mean values ± SD. The data shown are representative of results from at least three independent experiments performed with cells from at least three different healthy donors. Statistical analysis included one-way ANOVA in **(C)**, **(D)**, **(H)**, two-way ANOVA in **(G)**, and unpaired two-tailed Student’s t-test in **(B, F)**. Multiple comparisons were made using Bonferroni’s correction. ns, no significant difference, **P < 0.01, ****P < 0.0001.

Initially, we engineered BCMA/CS1 bispecific TRuC-T cells to secrete IL-7 and CCL21 (**Figure 4A**). Flow cytometric analysis revealed that BCMA/CS1 scFvs were more abundantly expressed on CD8^+^ T cells than those on CD4^+^ T cells (**Figure 4B**). Subsequent ELISA assays verified that BC-7×21 TRuC-T cells produced a relatively greater amount of IL-7 and CCL21 compared with original BCMA/CS1 bispecific TRuC-T cells (**Figures 4C, D**). Besides, BC-7×21 TRuC-T cells had a greater decrease in CFSE MFI and more absolute cell number counts than B-3G-C-3E TRuC-T cells, indicating a faster proliferation (**Figures 4E–G**). The CCL21 secreted by BC-7×21 TRuC-T cells possessed effective chemotaxis and could recruit more CFSE-labeled T cells (**Figures 4H, I**).

### BC-7×21 TRuC-T cells possessed superior persistence in multiple myeloma-bearing NCG mice

**Figure 5:**
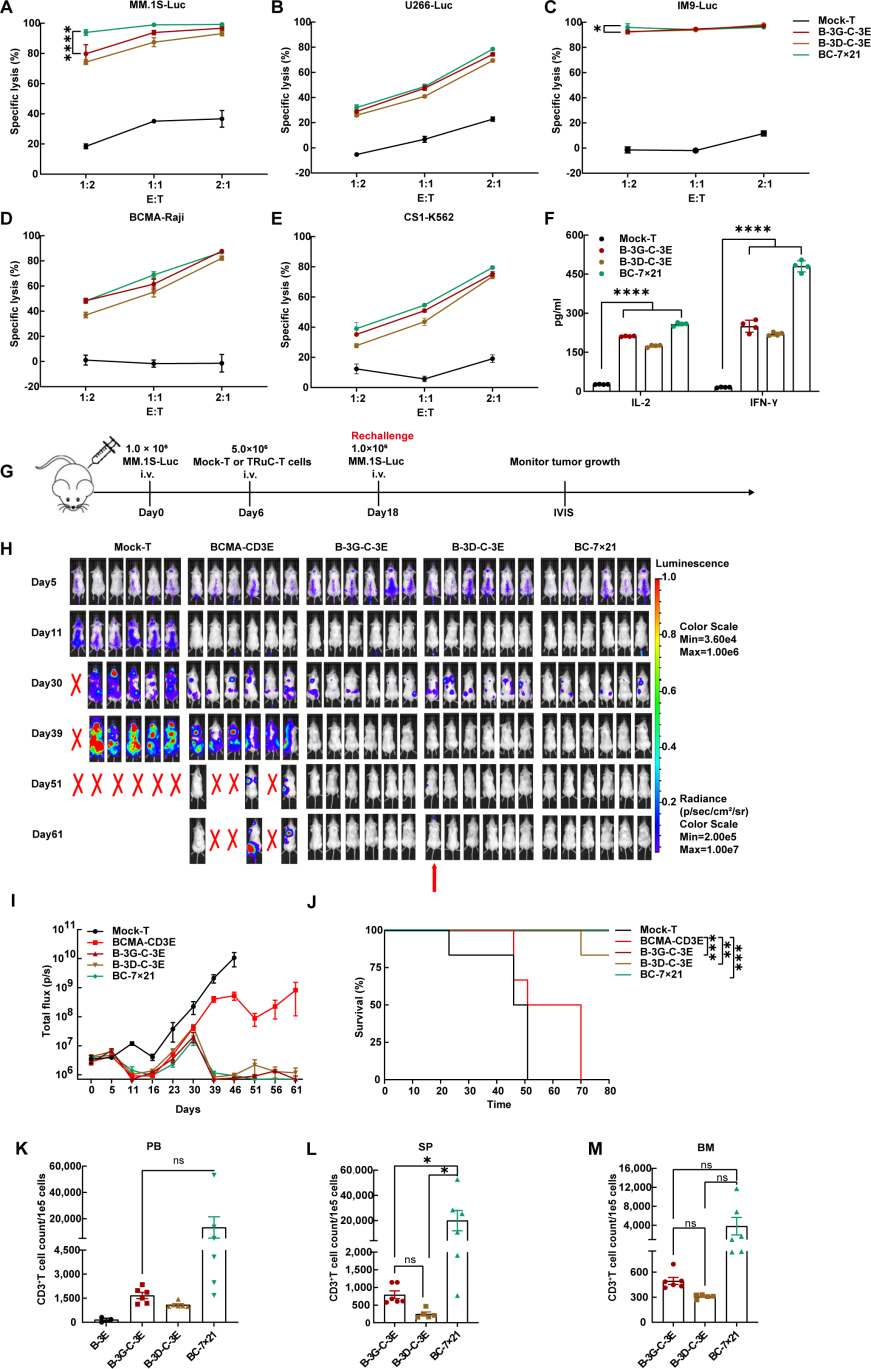
BC-7×21 TRuC-T cells acquired enhanced persistence *in vivo*. **(A–E)** Cytotoxic activity of BCMA/CS1 bispecific TRuC-T cells against MM.1S, U266, IM9, BCMA-Raji, and CS1-K562 cells at E: T ratios of 1:2, 1:1, and 2:1 for an 8 h coincubation (*N* = 3). **(F)** *In vitro* cytokine analysis of supernatants from co-culture of three different types of bispecific TRuC-T cells with U266 cells (E: T=1:1) for 24 h (*N* = 4). **(G)** Treatment scheme for MM.1S-luc tumor-bearing mice. **(H)** Tumor progression was monitored by bioluminescence imaging. The red arrow represented the mouse suffered from multiple myeloma recurrence. **(I)** Bioluminescence kinetics in each group of mice (*N* = 6). **(J)** Kaplan-Meier survival curve. Log-rank tests were used (*N* = 6). **(K)** The count of human CD3^+^ T cells in the peripheral blood from mice treated with BCMA-CD3E, B-3G-C-3E, B-3D-C-3E, and BC-7×21 TRuC-T cells on day 57. **(L, M)** The count of human CD3^+^ T cells in the spleen and bone marrow of mice in each group at the endpoint of treatment. Data are presented as mean values ± SD in **(A–F)** and mean values ± SEM in **(I)**, **(K–M)**. One-way ANOVA was used in **(F)**, **(K–M)**. *P*-values in **(A)** and **(C)** were calculated by two-way ANOVA. Multiple comparisons were made using Bonferroni’s correction. ns, no significant difference, *P < 0.05, **P < 0.01, ***P < 0.001, ****P < 0.0001.

BC-7×21 TRuC-T cells exhibited effective cytotoxicity against myeloma cell lines *in vitro*, and they outperformed B-3G-C-3E and B-3D-C-3E bispecific TRuC-T cells in killing MM.1S and IM9 cells at an E: T ratio of 1:2 (**Figures 5A–E**). After 24 h incubation with U266 cells (E: T=1:1), all bispecific TRuC-T cells secreted more IL-2 and IFN-γ than Mock-T cells (**Figure 5F**). NCG mice were engrafted with 1.0 × 10^6^ MM.1S-Luc cells on day 0. On day 6, the mice were randomly grouped to receive injection with 5.0 × 10^6^ cells from one of the following T cells: Mock-T, BCMA-CD3E, B-3G-C-3E, B-3D-C-3E, and BC-7 × 21 TRuC-T cells. Subsequent observations revealed a significant inhibition of multiple myeloma growth following the treatment with TRuC-T cells. The mice were rechallenged with 1.0 × 10^6^ MM.1S-Luc cells on day 18 (**Figure 5G**). It was shown that BCMA-CD3E TRuC-T cells exhibited inferior anti-tumor activity to B-3G-C-3E, B-3D-C-3E, and BC-7×21 TRuC-T cells, since 50% mortality occurred in this group on day 51. On day 61, a mouse from the B-3D-C-3E TRuC-T group suffered from a relapse and eventually died. In contrast, all mice in the B-3G-C-3E TRuC-T and BC-7×21 TRuC-T groups survived (**Figures 5H–J**). There was no significant change of body weight in each group of mice (**Supplementary Figure 3A**). Additionally, flow cytometric results showed that the BC-7×21 TRuC-T group mice had the highest levels of human CD3^+^T cells in their spleen (**Figures 5K–M**). Moreover, we tested the heart, liver, spleen, and kidney tissues from each group of mice with hematoxylin and eosin (H&E) staining, and no obvious inflammatory infiltrations were observed (**Supplementary Figure 3B**).

## Discussion

Although TCR is less expressed than CAR on T cell surface, TCR signaling is 10- to 100-fold more sensitive than CAR. TCR-induced signaling is slower but gentler than CAR and lasts longer (40, 41). Many studies have shown that enhancing TCR pathway signaling or stimulating alternative signaling pathways will further improve the function and differentiation of CAR-T cells *in vivo* and reduce tonic signaling caused by scFv-mediated CAR aggregation (42–44). The above may explain why TRuC- T cells could mediate competitive anti-tumor efficacy with CAR-T cells while reducing the production of some cytokines associated with CRS (17, 45). Recently, the first clinical trial of TRuC-T cell therapy has shown promising outcomes in refractory solid tumors (46). Therefore, we engineered TRuC-T cells targeting BCMA/CS1 by pairing two subunits of the TCR/CD3 complex to develop a more effective T cell therapy for multiple myeloma. Via systematic optimization, we generated BC-7×21 TRuC-T cells secreting IL-7 and CCL21 that could robustly eliminate multiple myeloma cells *in vitro* and *in vivo*. In addition to TCRαC and TCRβC, the N-terminus of CD3γ, CD3δ, and CD3ϵ subunits of the TCR/CD3 complex could be fused to BCMA or CS1 scFv using a linker sequence. All three BCMA or CS1-specific TRuCs were integrated into the TCR/CD3 complex and CD3ϵ-TRuC showed the highest MFI of scFvs, in accordance with αβTCRs harboring two CD3ϵ subunits (14, 15). Similarly, our results showed that the bispecific TRuCs of CD3γ-CD3ϵ and CD3δ-CD3ϵ were able to integrate into the endogenous TCR/CD3 complex with high expression efficiency on the surface of T cells. Previous research highlighted ϵ-TRuC (scFv fused to CD3ϵ) T cells as the most effective (16), yet our findings indicate that only the CD3ϵ-TRuC at the upstream of the expression vector was detectable on the surface of T cells in the constructed CD3ϵ-CD3ϵ panel. Moreover, after 16 days of *in vitro* culture, the expression rates of B-3D-C-3E and C-3D-B-3E TRuCs on T cells decreased, with the emergence of single-positive T cells. This outcome suggests that the fusion protein constituted by the CD3δ subunit is not stably integrated into the TCR/CD3 complex, as the isolated TCR subunit fails to be effectively transported and expressed on the surface of T cells (47).

Safety is one of the major challenges, including precise tumor targeting to avoid off-target or on-target/off-tumor toxicity (48, 49). Lymphocytes (including B, T, and natural killer cells) express CS1 to a lower extent than multiple myeloma cells (35, 36). Previous study has found that TCR is a self-restrained signaling machinery owing to mono-phosphorylation of CD3ϵ ITAMs subpopulation (50), suggesting that CS1 scFv-CD3ϵ panel may work better. The proportion of CD8^+^ T cells in anti-CS1 CAR-T cells was actually lower than that of CS1-CD3G, CS1-CD3D, and CS1-CD3E TRuC-T cells, indicating lower fratricide propensity by anti-CS1 TRuC-T cells than anti-CS1CAR-T cells. Given the unique characteristics of TCR/CD3 subunits and CS1’s expression on CD8^+^ T cells, four bispecific BCMA/CS1 TRuC-T cells had been developed. We found that C-3G-B-3E and C-3D-B-3E TRuC-T cells had fewer CD8^+^T cells and exhibited increased expression of immune checkpoints, with the C-3D-B-3E TRuC-T cells having poor survivability. Although no serious toxicity or side effects were observed with CS1 monoclonal antibody (51), we could integrate the suicide molecule as a safety switch into our design (52).

In our *in vitro* cytotoxicity assays, we observed B-3G-C-3E TRuC-T cells exhibited better killing ability at an E: T ratio of 1:1. Our NCG mouse xenograft model also demonstrated that B-3G-C-3E TRuC-T cells owned a stronger tumor-killing capacity than B-3D-C-3E TRuC-T cells. Accordingly, the recurrence was detected in the B-3D-C-3E TRuC-T group. Recurrence of multiple myeloma may stem from various factors, such as loss of BCMA expression or the residual of resistant multiple myeloma cells (53–55). We found that the relapsed tumor cells retained antigen expression, but the intensity of BCMA antigen expression decreased compared to the Mock-T group. This result indicated that BCMA may be particularly susceptible to antigen escape under selective pressure from TRuC-T-cell therapy (**Supplementary Figure 2E**). Furthermore, the failure to eradicate tumors was likely attributable to the low T-cell dose administered which was potentially related to the unstable integration of B-3D-C-3E TRuCs on T cells surface. For better *in vivo* efficacy, TRuC-T cell dose exploration is needed.

Deficiency in CAR-T cell persistence has also been identified as another factor contributing to recurrence in patients with multiple myeloma. IL-7 is a potent immune regulatory protein (56), and thus necessary for proliferation and survival of naïve and mature T cells (57–59). Accordingly, IL-7 was demonstrated to increase the proliferative capacity of BC-7x21 TRuC-T cells. Moreover, our group have reported that BCMA or CD19 CAR-T cells expressing IL-7 and CCL19 may represent a promising therapy for relapsed/refractory multiple myeloma or B cell malignancies (33, 60). Similar to CCL19, CCL21 is also a ligand of the homeostatic chemokine for human CC chemokine receptor 7(CCR7), belonging to one of the G protein-coupled receptor family which is absolutely required for the directional migration of immune cells into the T cell zone (61–63). CCL21 secreted by BC 7x21 TRuC-T cells possessed effective chemotaxis and recruit more CFSE-labeled T cells. For the rechallenged model, there was a good response in the early stages of single BCMA-CD3E TRuC-T cell treatment while the multiple myeloma relapsed in the later stages. Dual TRuC, which targets both BCMA and CS1 simultaneously, provided better inhibition of tumor relapse. Despite enhanced TRuC-T persistence in mice, the survival of BC-7×21 TRuC-T cell treated mice was not significantly better in the severely immunodeficient mouse model, that lacks inherent environment of human immune system, compared with B-3G-C-3E bispecific TRuC-T cells. Due to the absence of costimulatory signals, the first-generation CARs containing scFvs and an intracellular CD3ζ domain exhibit limited proliferative capacity and anti-tumor effects (64). To improve the anti-tumor activity and persistence of TRuc-T cells, we can also provide additional costimulatory domains (65).

In this work, we took advantage of the integration of TRuC into TCR/CD3 signaling to design bispecific TRuC-T cells by targeting both BCMA and CS1. We systematically compared five different subunits of TCR/CD3 complex and found the superiority of CD3γ-CD3ϵ and CD3δ-CD3ϵ panels. BC-7×21 TRuC-T cells exhibited stronger proliferation, chemotaxis and cytolytic activity, highlighting the importance of additional secretion of cytokines. In conclusion, we have presented a rational approach for the engineering of BCMA/CS1 bispecific TRuC-T cells that can effectively target multiple myeloma and substantially reduce the probability of tumor antigen escape. BCMA/CS1 bispecific TRuC-T cells secreting IL-7 and CCL21 may represent a novel therapy for relapsed/refractory multiple myeloma.

## Data Availability

The raw data supporting the conclusions of this article will be made available by the authors, without undue reservation.
