# Supplementary material for: Optimized BCMA/CS1 bispecific TRuC-T cells secreting IL-7 and CCL21 robustly control multiple myeloma
Source: Front Immunol. 2024 Dec 24;15:1502936. doi: 10.3389/fimmu.2024.1502936 (PMC11703830; doi:10.3389/fimmu.2024.1502936)
Supplement: Supplementary file 1 [file DataSheet1.docx]

Supplementary Material


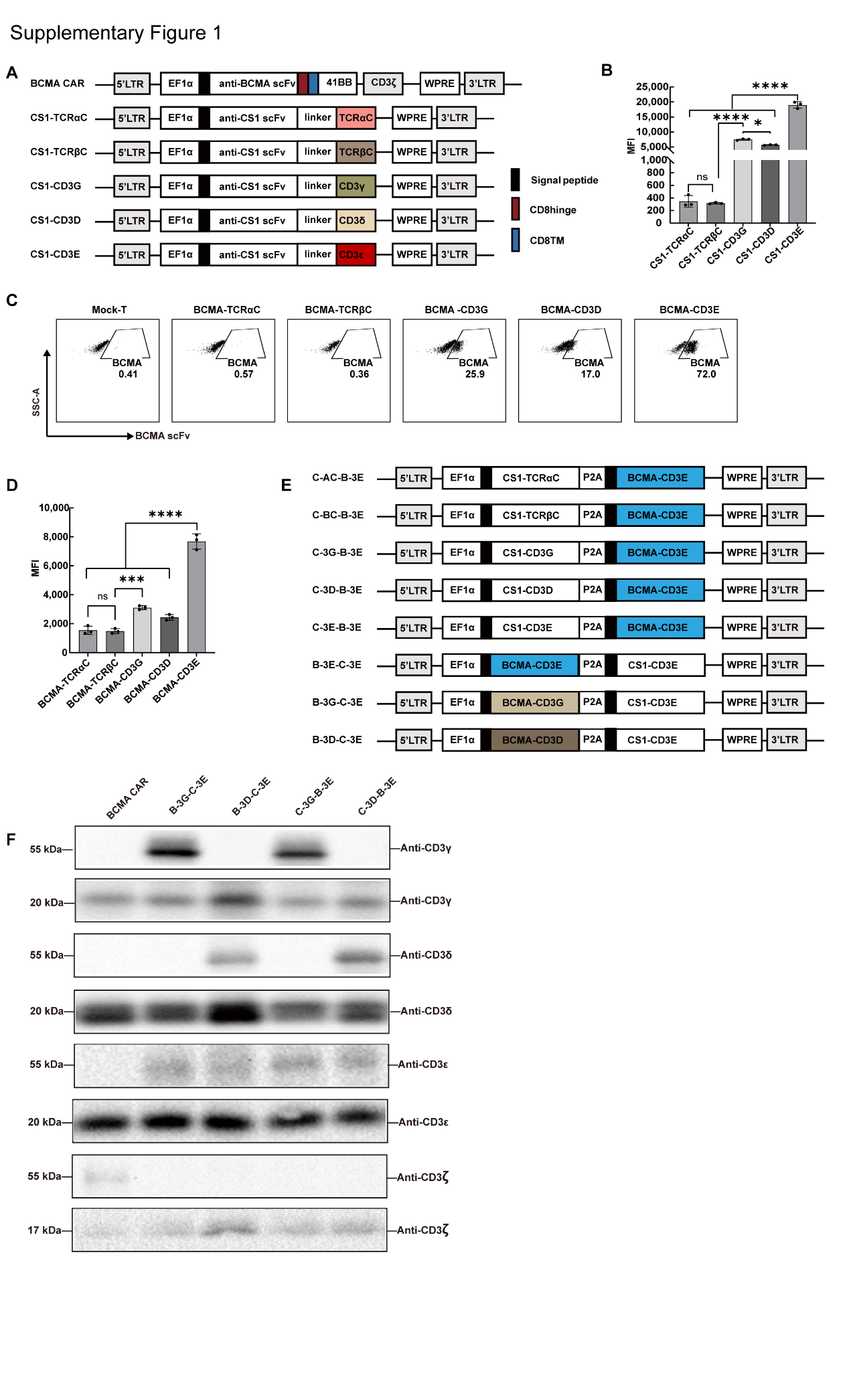


**Supplementary Figure 1.** Construction of BCMA or CS1 single-target and BCMA/CS1 bispecific TRuC-T. **A.** Schematic illustration of CS1 single-target TRuCs. **B.** MFI of CS1 single-target TRuCs (*N* = 3). **C and D.** The expression rates and statistics of BCMA single-target TRuCs (*N* = 3). **E.** Schematic illustration of BCMA/CS1 bispecific TRuCs. **F.** Exogenous 55KDa TRuC fusion protein and endogenous 20 KDa CD3γ, CD3δ, CD3ε, and 17 KDa CD3ζ were detected by western blot. Data are shown as mean values ± SD. *P*-values in (**B) and** (**D)** were calculated by one-way ANOVA. Multiple comparisons were made using Bonferroni’s correction. ns, no significant difference, **P* < 0.05, ****P* < 0.001, *****P* < 0.0001.


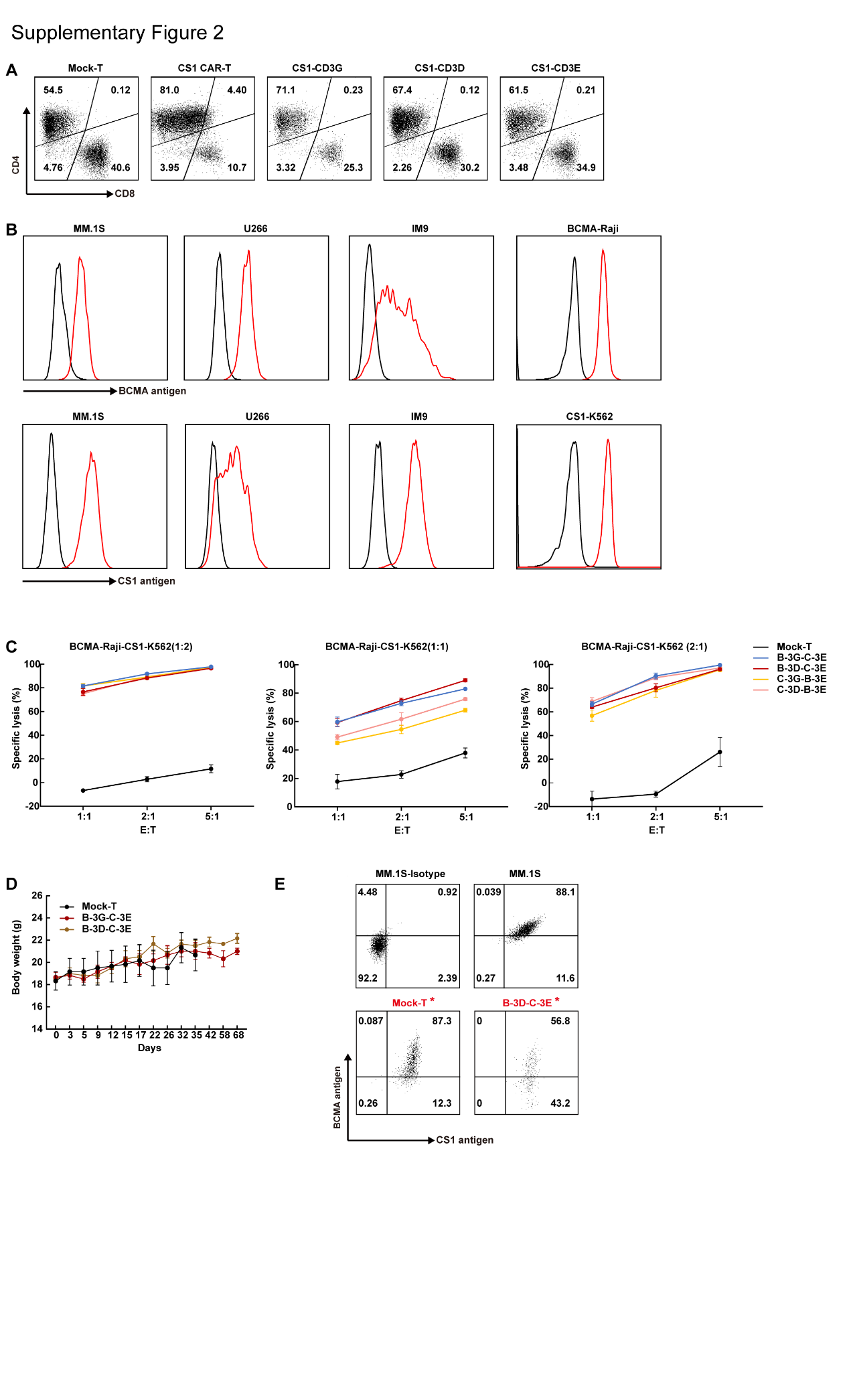


**Supplementary Figure 2.** Killing ability of bispecific TRuC-T cells against BCMA-Raji and CS1-K562 cells**. A.** CD4^+^ T and CD8^+^ T ratios of anti-CS1 CAR-T and anti-CS1 TRuC-T cells. **B.** BCMA or CS1 antigen expression level on the surface of MM.1S, U266, IM9, BCMA-Raji, and CS1-K562 cells. **C**. Cytotoxic activity of BCMA/CS1 bispecific TRuC-T cells against BCMA-Raji and CS1-K562 cells at E: T ratios of 1:1, 2:1, and 5:1 for an 8 h coincubation. BCMA-Raji and CS1-K562 were mixed according to the ratios of BCMA-Raji: CS1-K562=1:2, BCMA-Raji: CS1-K562=1:1, and BCMA-Raji: CS1-K562=2:1 (*N* = 3). **D.** Changes of body weight in each group of mice (*N* = 3). **E.** BCMA and CS1 antigen expression level on multiple myeloma cells isolated from the bone marrow of relapsed mice. *Data shown are tumors isolated from mice treated with Mock-T cells and B-3D-C-3E TRuC-T cells.


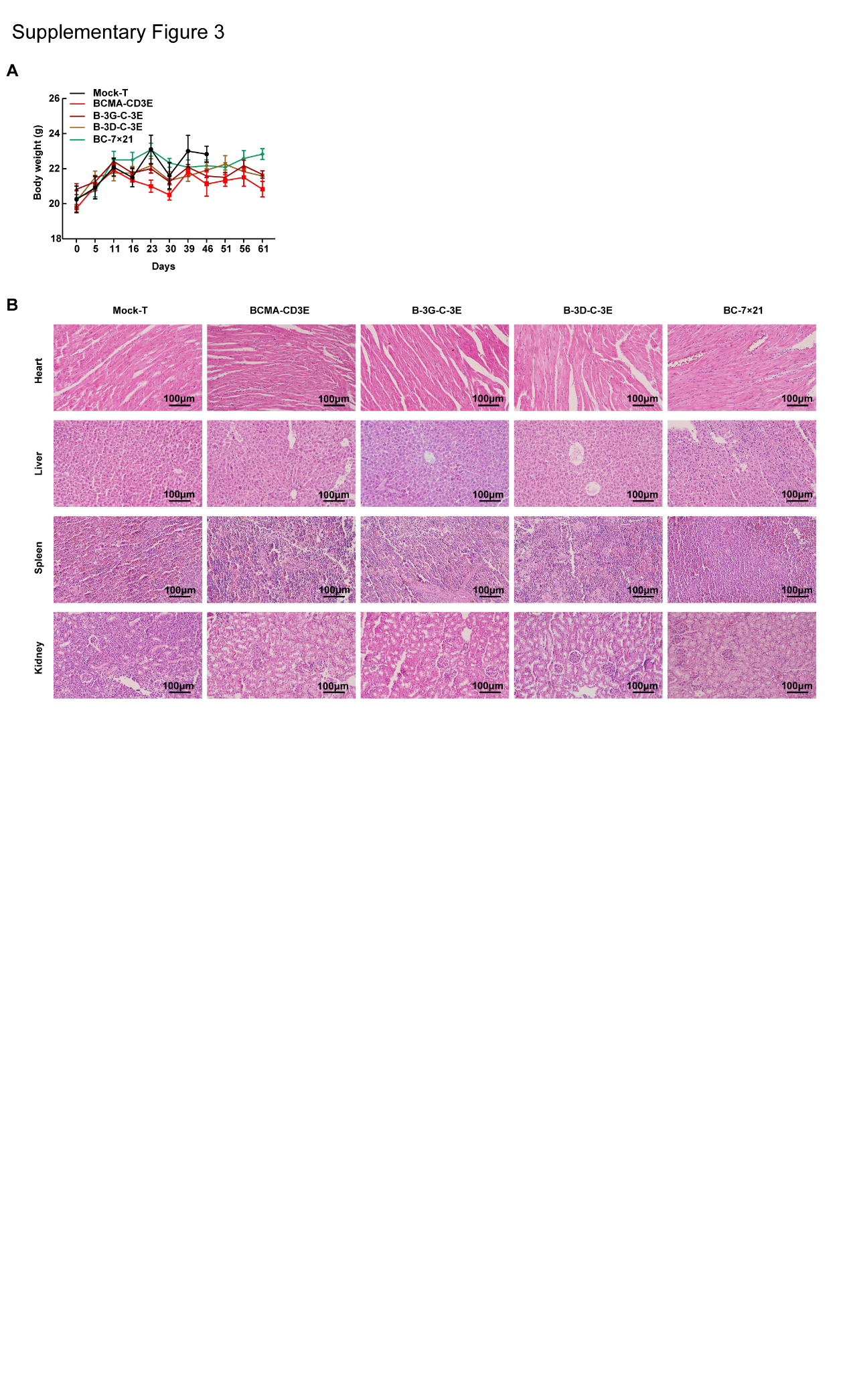


**Supplementary Figure 3.** No obvious pathological changes were observed in the tissues of the mice. **A**. Changes in body weight among each group of mice (*N* = 6). **B**. The heart, liver, spleen, and kidney tissues of the Mock-T, BCMA-C3E, B-3G-C-3E, B-3D-C-3E, and BC-7×21 TRuC-T cell treatment groups were examined using hematoxylin and eosin (H&E) staining
